# Efficient Multi-Task Training with Adaptive Feature Alignment for Universal Image Segmentation

**DOI:** 10.3390/s25020359

**Published:** 2025-01-09

**Authors:** Yipeng Qu, Joohee Kim

**Affiliations:** Department of Electrical and Computer Engineering, Illinois Institute of Technology, Chicago, IL 60616, USA; yqu13@hawk.iit.edu

**Keywords:** computer vision, universal image segmentation, multimodal learning, feature alignment

## Abstract

Universal image segmentation aims to handle all segmentation tasks within a single model architecture and ideally requires only one training phase. To achieve task-conditioned joint training, a task token needs to be used in the multi-task training to condition the model for specific tasks. Existing approaches generate the task token from a text input (e.g., “the task is panoptic”). However, such text-based inputs merely serve as labels and fail to capture the inherent differences between tasks, potentially misleading the model. In addition, the discrepancy between visual and textual modalities limits the performance gains in existing text-involved segmentation models. Nevertheless, prevailing modality-alignment methods rely on large-scale uni-modal encoders for both modalities and an extremely large amount of paired data for training, and therefore it is hard to apply these existing models to lightweight segmentation models and resource-constrained devices. In this paper, we propose Adaptive Feature Alignment (AFA) integrated with a learnable task token to address these issues. The learnable task token automatically captures inter-task differences from both image features and text queries during training, providing a more effective and efficient solution than a predefined text-based token. To efficiently align the two modalities without introducing extra complexity, we reconsider the differences between a text token and an image token and replace image features with class-specific means in our proposed AFA. We evaluate our model performance on the ADE20K and Cityscapes datasets. Experimental results demonstrate that our model surpasses baseline models in both efficiency and effectiveness, achieving state-of-the-art performance among segmentation models with a comparable amount of parameters.

## 1. Introduction

Image segmentation aims to interpret an image by segmenting different objects from each other presented in the image. Based on segmentation principles, three different segmentation tasks are defined. Semantic segmentation classifies each pixel by category, and instance segmentation classifies each pixel, while also distinguishing between different instances of the same object class. Panoptic segmentation combines both semantic and instance segmentation to provide a more comprehensive understanding of an image.

To unify these tasks within a single network architecture, MaskFormer [1,2] treats image segmentation as a mask classification problem. It introduces a transformer decoder module from DETR [3] and uses object queries to progressively refine region proposals for final prediction. However, this approach requires multiple training cycles to train the model for each segmentation task separately. To address this limitation, OneFormer [4] enhances Mask2Former [2] by introducing a task-conditioned joint training scheme. This method helps the model adapt to different tasks by conditioning object queries with a task token generated from a predefined text input: *“the task is {TASK}"*, where *{TASK}* is uniformly sampled from *{semantic, instance, panoptic}* and assigned to each image during training. However, this predefined task input merely provides the task name and fails to capture the intrinsic distinctions between different segmentation tasks. In addition, the predefined task token for a specific task remains constant after training, leading to unnecessary computational overhead during evaluation. To address these issues, we propose the use of learnable task tokens that offer several advantages over predefined ones. First, a learnable task token can acquire comprehensive and robust representations of cross-task distinctions beyond what a predefined task token can capture, as it can be updated automatically through interactions with image and text modalities during training. Second, learnable task tokens can be directly utilized during inference without the need for generation, thereby reducing computational costs and improving efficiency. To implement this, we define a set of learnable parameters in our model to replace the predefined task tokens, with each parameter corresponding to one of the segmentation tasks. Experimental results demonstrate that this straightforward approach of using learnable task tokens significantly enhances the model’s performance, particularly in semantic segmentation.

To incorporate text supervision into image segmentation, OneFormer [4] integrates a query–text contrastive loss between object queries and text queries using a fixed one-to-one matching mechanism. In this step, text supervision is extracted from ground-truth labels to produce the text queries. However, the text-based supervision in OneFormer [4] involves significant redundancy due to padding operations. To address this, our previous work, EQO [5], redesigns the text template for extracting supervisory information and proposes an attention-based contrastive loss, achieving parameter and computation efficiency. Despite the improvements made by EQO [5], cross-modality differences remain between the two branches of the model throughout the training stage. For example, in [4,5], the image branch consists of a complete universal image segmentation model, while the text branch includes a uni-modal encoder that processes the textual modality only. The query–text contrastive loss is computed without aligning features from the two modalities. Recent studies [6,7] have shown that learning a joint embedding space across different modalities can enhance performance in downstream vision tasks. Motivated by this, we hypothesize that aligning the two modalities in EQO [5] could further improve its performance. However, existing methods [6,8,9] for modality alignment often rely on large-scale, computationally intensive encoders and vast image-paired training data, which are not friendly to lightweight segmentation models trained on resource-constraint devices. To achieve efficient alignment without these resources, we address the disparity in information density between image tokens and text tokens that can necessitate significant computational costs and extensive training data for alignment. Specifically, we utilize ground-truth binary masks to compute class-specific means over foreground regions, and replace the original image features in the alignment process. This approach facilitates efficient and effective modality alignment in a segmentation model without introducing extra complexity.

We name our proposed framework as **Adaptive Feature Alignment** (AFA), a novel approach to address the limitations of existing image segmentation models that utilize text supervision. A learnable task token is integrated with AFA that simultaneously captures cross-task differences from both image features and textual supervision. Furthermore, AFA reinterprets the cross-modality differences within text-guided segmentation models and presents an efficient and effective modality alignment method by leveraging the semantic equivalence between text queries and class-specific means.

Our major contributions are summarized as follows:

- **Identifying Limitations in Text-Supervised Universal Image Segmentation Models**: We reveal the limitations of universal image segmentation models that utilize text supervision. Specifically, predefined text inputs offer limited guidance on inter-task differences for generating task tokens. Moreover, the cross-modality barrier between the image and text branches of the model makes it challenging to effectively learn from text supervision.
- **Proposing Adaptive Feature Alignment (AFA)**: We introduce Adaptive Feature Alignment (AFA), which incorporates a learnable task token to achieve cross-modality alignment in text-supervised image segmentation. This approach enhances adaptability to different segmentation tasks and improves both segmentation precision and computational efficiency.
- **Demonstrating Superior Performance through Comprehensive Evaluation**: We evaluate our model across three segmentation tasks using two datasets—ADE20K [10] and Cityscapes [11]. Our model surpasses its meta-architecture [5] while achieving gains in model efficiency and computational complexity reduction. Compared to other universal segmentation models of similar size, our model exhibits even greater performance advantages. Specifically, on the ADE20K dataset, we achieve **44.4 PQ** in panoptic segmentation, **50.1 mIoU** in semantic segmentation, and **29.3 AP** in instance segmentation. On the Cityscapes dataset, our approach achieves **81.0 mIoU** (single-scale), **42.4 AP**, and **65.3 PQ**. Additionally, we conduct extensive and detailed analyses of our approach.

The remainder of this paper is organized as follows. Section 2 reviews recent works closely related to ours. Section 3 details our proposed approach. Section 4 presents the experimental results and analyses of our model. Section 5 discusses the limitations of our approach. Finally, we conclude the paper and summarize its contributions in Section 6.

## 2. Related Work

**Universal Image Segmentation.** A universal segmentation model aims to perform semantic, instance, and panoptic segmentation tasks using a single network. The MaskFormer framework [1,2] achieves this by treating all segmentation tasks as mask-classification problems. However, it still requires multiple training runs, each dedicated to a specific segmentation task. To address this limitation, OneFormer [4] introduces a multi-task training strategy that enables universal segmentation with a single training cycle. Building upon OneFormer, EQO [5] identifies redundancy in its text supervision. It presents an efficient text template to extract text-based supervisory information from visual annotations. Additionally, EQO introduces an attention-based contrastive loss that supports one-to-many matching between text queries and object queries. This loss enhances performance by aligning with the nature of object queries, where one query can capture objects from multiple categories. Despite these advancements, cross-modality discrepancies and the use of a vague predefined task token continue to hinder further performance improvements. To address these challenges, our proposed method aims to align the modalities and reinforce inter-task distinctions during training.

**Cross-Modality Alignment.** Cross-modality alignment is presented to learn a joint embedding space between two different modalities. CLIP [8] processes image–text pairs using two separate encoders to obtain image and text embeddings. Contrastive learning is then applied between these two sets of embeddings, with the objective of aligning the two modalities by maximizing the similarity between each image embedding and its corresponding text embedding. This strategy enables the pre-trained model to adapt effectively to downstream tasks, even in zero-shot and open-vocabulary scenarios. Following this approach, CoCa [12] aims to enhance the performance of image–text pre-training by introducing an image captioning loss, further strengthening the alignment between images and textual descriptions. FILIP [13] achieves finer-grained alignment by maximizing the token-wise similarity between the two modalities, allowing for more detailed correspondence between image regions and words. Numerous other works [8,9,14,15,16,17,18,19] have continued to contribute to this area by exploring various strategies for multimodal representation learning. Furthermore, some methods [6,7] extend the concept of image–text alignment to bind any two modalities using contrastive learning. These approaches aim to learn a joint embedding space that accommodates multiple modalities, thereby facilitating cross-modal understanding and interaction. Motivated by these works, we believe incorporating modality alignment has the potential to further enhance the performance of a segmentation model with text supervision. However, most of these approaches require large-scale encoders and web-scale image-paired data. For instance, in [6], an image encoder utilizes a ViT-H architecture [20] with 630 million parameters and a text encoder comprising 302 million parameters [21]. Such approaches are impractical for lightweight segmentation models with text supervision. To address this limitation, we propose an approach to achieve efficient cross-modality alignment.

**Image Segmentation with Text Supervision.** Recent research has explored various strategies for improving image segmentation with text supervision. One approach is open-vocabulary segmentation [22,23,24], which leverages pre-trained vision–language models by fine-tuning on manually annotated images. Another line of work focuses on using text supervision solely during training [25,26]. On the other hand, UniLSeg [27] fuses images and text prompts in a joint embedding space for universal segmentation, while OMG-Seg [28] unifies multiple segmentation tasks—including image, video, and open vocabulary—in a shared decoder. Building on OMG-Seg, OMG-LLaVA [29] integrates reasoning across image, object, and pixel levels. Meanwhile, diffusion-based methods [30,31,32] have attracted attention for generating high-resolution images from text prompts, with several studies [33,34], adapting them for segmentation. In contrast, OneFormer [4] and EQO [5] neither employ pre-trained vision–language models nor use external data, deriving text supervision solely from ground-truth labels. However, their frameworks process text and images in separate branches and compute the query–text contrastive loss without modality alignment. To address this, we propose binding the two branches in a parameter- and computation-efficient manner.

## 3. Proposed Method

### 3.1. Preliminaries

**Figure 1:**
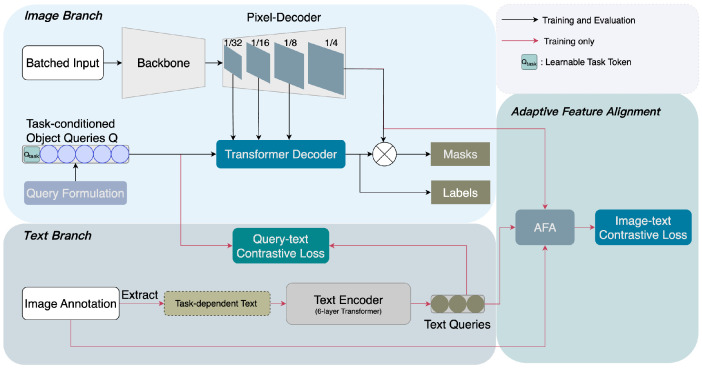
Architecture Overview. We propose Adaptive Feature Alignment (AFA) integrated with a learnable task token to address existing issues in our baseline models [4,5]. First, AFA effectively achieves cross-modality alignment without increasing model complexity. Second, the query formulation module to construct task-conditioned object queries is driven by our proposed learnable task token, enhancing the model’s adaptability across different segmentation tasks.

**Figure 2:**
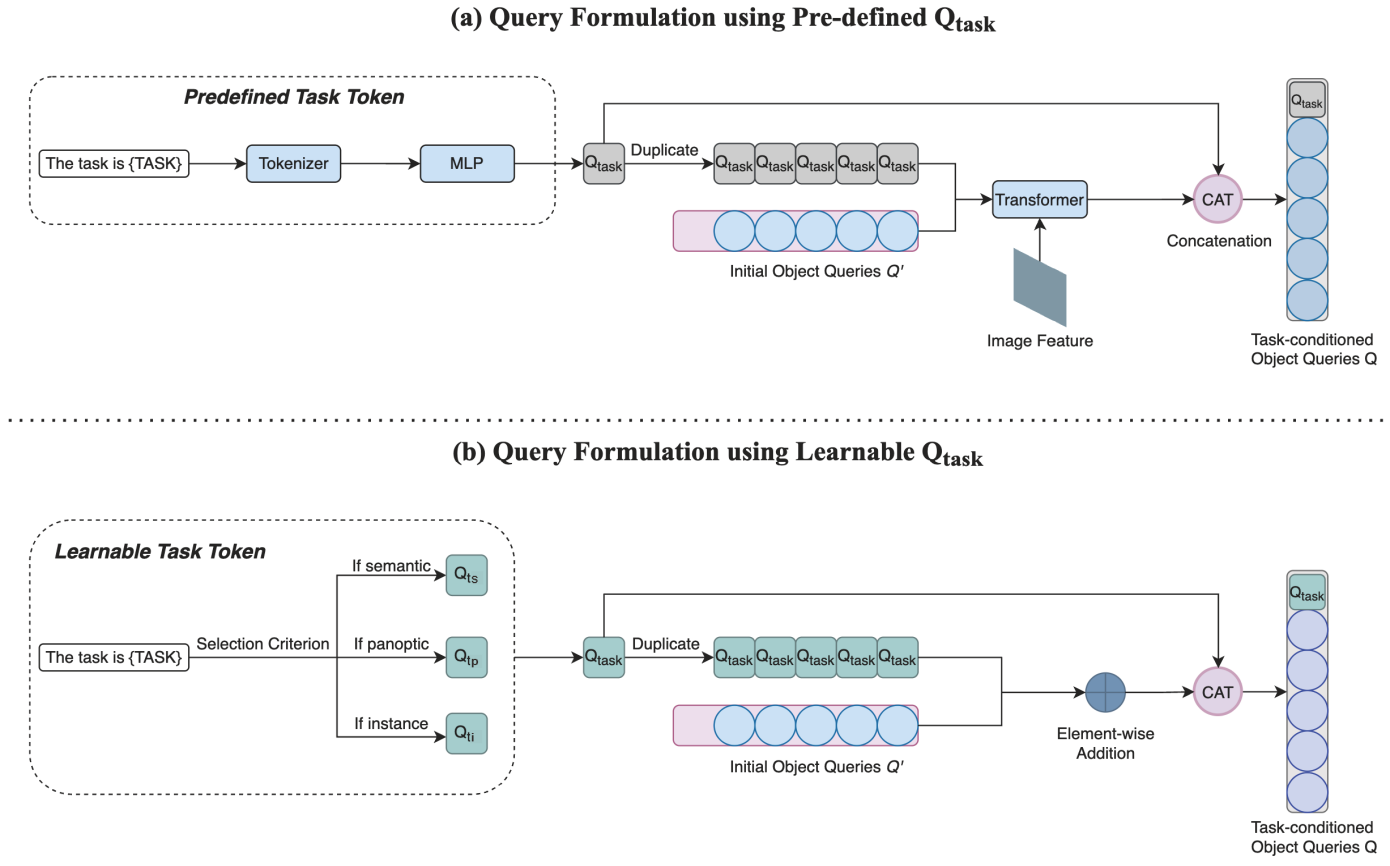
We compare our query formulation approach using a learnable task token with that of our baseline models [4,5]. Our task token is defined as a group of learnable parameters, and the original text input *“The task is {TASK}"* is retained but used as a selection criterion only. Different from the query formulation with predefined $Q_{task}$, we discard the tokenizer, MLP, the transformer module, and the involvement of image features produced by the pixel decoder to achieve efficiency gains. The element-wise addition and concatenation are applied to generate task-conditioned object queries, which are fed into the transformer decoder module to produce predictions and perform query–text contrastive learning with text queries.

Before introducing our approach, we first review the meta-architecture EQO [5], upon which our model is built. EQO comprises four main components:

(1) The **encoder–decoder feature extractor** includes a Swin-T backbone [35,36] to extract multi-scale feature maps from the input image, and a pixel decoder to progressively upsample these feature maps for producing detailed and high-resolution representations. This corresponds to the backbone and the pixel decoder of our model as shown in the image branch of Figure 1.
(2) The **task-conditioned query formulation module** initializes object queries as the repetitions of a task token generated from a predefined text input. This conditions the queries based on the specific segmentation task at hand. For each image fed into the model, object queries are specialized with the extracted image features in a two-layer transformer decoder. Our method simplifies this procedure by leveraging a learnable task token, and the comparison between two different query formulation methods is depicted in Figure 2.
(3) **Efficient query optimizer** performs query–text contrastive learning to reinforce the inter-class distinctions. After extracting text supervision from the ground-truth masks, EQO processes the text tokens to produce text queries with a text encoder [25]. An attention-based contrastive loss is then applied to measure the similarity between the object queries and text queries, using a one-to-many matching mechanism. We adopt this design to generate text queries and compute query–text contrastive loss in our model as shown in the text branch of Figure 1.
(4) The **prediction head** includes a DETR [37] decoder, which is used to obtain the task-dynamic class and mask predictions. This corresponds to the transformer decoder module in our model.

### 3.2. Learnable Task Token for Efficient Universal Image Segmentation

In universal image segmentation models [4,5], a task token is employed to condition the model to train for a specific segmentation task. This token is generated from a predefined text template *“the task is {TASK}”*. Subsequently, the predefined task token interacts with image features produced by the pixel decoder to formulate task-conditioned object queries as illustrated in Figure 2a.

However, this procedure introduces computational redundancy and results in uninformative task tokens. To address these issues, we propose a straightforward approach in which the task token is selected from a set of learnable parameters, each corresponding to one of the segmentation tasks. A learnable task token can automatically acquire the inter-task difference from image and text modalities through the training stage. The predefined text input for the original task token is still retained but used solely as a selection criterion. The detailed procedure is presented in Algorithm 1. We first initialize 3 learnable parameters, each corresponding to one segmentation task. After determining which task is assigned, the corresponding learnable task token $Q_{task}$ is selected and duplicated N−1 times, where *N* is a hyperparameter denoting the total number of object queries. Element-wise addition is then applied between the duplicated task tokens $Q_{task}^{dup}$ and initial N−1 object queries. Finally, the learnable task token $Q_{task}$ is concatenated with the previous addition result to formulate *N* task-conditioned object queries.
**Algorithm 1** Pseudocode of query formulation using a learnable task token.**Learnable Params** $Q_{t}\in R^{3\times dim}$
▹ “dim” equals the number of channels
$Q_{ti}\in R^{1\times dim}$
▹ One row of $Q_{t}$; i∈{s,p,i}
{TASK}←{semantic,instance,panoptic}
▹ Random sampling
$Q^{\prime }\in R^{(N-1)\times dim}$
▹$Q^{\prime }$: N−1 initial object queries;


▹ “N” is the total number of object queries
**if** {TASK} == “semantic” **then**

   
$Q_{task}\leftarrow Q_{ts}$

▹$Q_{task}$: the learnable task token
**else if** {TASK} is “panoptic” **then**

   
$Q_{task}\leftarrow Q_{tp}$

**else**
   
$Q_{task}\leftarrow Q_{ti}$

**end if**
$Q_{task}^{dup}\in R^{(N-1)\times dim}\leftarrow Duplicate\left\{Q_{task}\right\}$
$Q\leftarrow cat(Q_{task},Q^{\prime }+Q_{task}^{dup})$
▹ “cat”: Concatenation
**return** $Q\in R^{N\times dim}$
▹ Q: task-conditioned object queries


Compared to the query formulation using the predefined task token [4,5], we eliminate the transformer module previously used to specialize object queries with image features to further enhance the model efficiency as illustrated in Figure 2b. Experimental results demonstrate that the updated model achieves higher accuracy than before. This outcome not only underscores the effectiveness of our proposed learnable task token but also suggests that incorporating image features into query formulation is redundant.

### 3.3. Adaptive Feature Alignment for Efficient Cross-Modality Learning

In universal image segmentation models that incorporate text queries [4,5], the query–text contrastive loss is computed without adequately addressing the cross-modality gap between the image and text branches. This limitation hampers the model’s ability to effectively learn from text supervision. However, existing methods for modality alignment often rely on large-scale and computationally intensive uni-modal encoders. Such resource-intensive methods are impractical for lightweight segmentation models trained with manually annotated images. To overcome this limitation and achieve efficient modality alignment, we revisit the basic units of the two modalities in segmentation models [4,5] that use text supervision. We identify that the information density differs between image tokens (from the pixel decoder) and text tokens (from the text encoder). Each text token contains class-level semantic information, while an image token includes pixel-level details. We hypothesize that this disparity complicates the modality alignment process and necessitates large-scale encoders trained with extensive data. To validate our assumption, we propose Adaptive Feature Alignment (AFA), which aims to effectively bind image features and text queries in a parameter- and computation-efficient manner.

**Figure 3:**
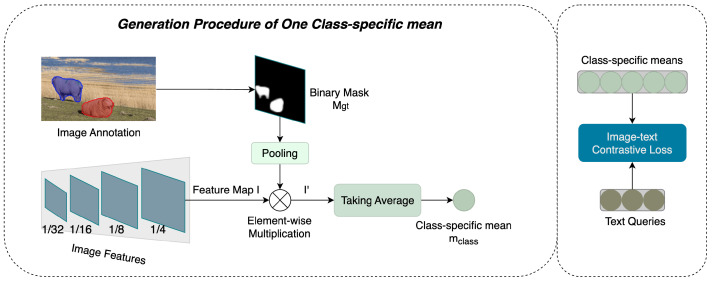
AFA. Using one ground-truth binary mask of an image, AFA temporarily makes the image tokens of a feature map *I* equal to zero if they are not located in the foreground regions. Then, AFA can take the average of all remaining tokens and obtain the class-specific mean. If there are *M* binary masks for the image, such a procedure would be repeated for *M* times to produce *M* class-specific means. The image–text contrastive loss [5] is computed between the resulting class-specific means and text queries from the text branch.

The core idea of AFA is to ensure symmetry between the two alignment subjects in terms of sequence length and, more importantly, information density. To achieve this, we aim to extract class-specific means from an image feature map “I” to serve as counterparts to the text queries, where each extracted item encapsulates the semantics of a single object class. In the pixel decoder, the feature map *I* is used to generate the final mask predictions, so the localization information of foreground objects is consistent between the ground-truth masks and *I*. Therefore, we first utilize a ground-truth mask $M_{gt}$ to identify the corresponding regions of instances present in *I*. This allows us to extract all relevant image tokens from the feature map *I*. As illustrated in Figure 3, for objects of a particular class in an image, we identify the foreground regions and extract all the image tokens within these regions by multiplying the feature map *I* with the binary mask $M_{gt}$. To reduce the computational complexity in computing the image–text contrastive loss and to address the sequence length disparity between text queries and extracted image tokens, we aim to merge these tokens into a single representation. ALGM [38] found that averaging similar image tokens yields better performance than other merging methods. Inspired by this, we aggregate the extracted image tokens by computing their average. The resulting output, referred to as the class-specific mean, contains an equivalent amount of semantic information as a text query. For each ground-truth mask, a class-specific mean is produced accordingly. The overall procedure is shown in Algorithm 2.
**Algorithm 2:** Pseudocode of producing a class-specific mean.**Ground-truth Mask:** $M_{gt}\in R^{H^{\prime }\times W^{\prime }}$
▹$H^{\prime },W^{\prime }$: the resolution of images
**Image Feature Map:** $I\in R^{C\times H\times W}$
▹H,W: resolution of the feature map


▹ *C*: the number of channels
$M_{pooled}\in R^{H\times W}\leftarrow pooling\left(M_{gt}\in R^{H^{\prime }\times W^{\prime }}\right)$
$I^{\prime }\in R^{C\times H\times W}\leftarrow I⊗M_{pooled}$
▹⊗: element-wise multiplication
**Class-specifc mean:** 
$m_{class}\leftarrow sum\left(I^{\prime }\right)/sum\left(M_{pooled}\right)$

**return** 
$m_{classs}\in R^{C}$



By computing the attention-based contrastive loss [5] between the class-specific means and the text queries, we effectively align image features with text queries. This alignment enables object queries from the image branch to learn more comprehensive representations through the original query–text contrastive learning.

In addition to the contrastive loss ($L_{contra}$), we also calculate the classification CE-loss ($L_{cls}$), dice loss ($L_{dice}$), and binary cross-entropy loss ($L_{bce}$).The final loss computation is a weighted sum of four losses as shown in Equations (1) and (2). Following [4,5], we set $\lambda_{contra}=0.5$, $\lambda_{cls}=2$, $\lambda_{dice}=5$, $\lambda_{bce}=5$. Specifically, the contrastive loss $L_{contra}$ equals the sum of the image–text contrastive loss $L_{m_{class}↔Q_{text}}$ and the query–text contrastive loss $L_{Q↔Q_{text}}$:

(1) $$\begin{array}{cc} L_{final}\; & =\lambda_{contra}L_{contra}+\lambda_{cls}L_{cls}+\lambda_{dice}L_{dice}+\lambda_{bce}L_{bce} \end{array}$$

(2) $$\begin{array}{cc} L_{contra}\; & =L_{m_{class}↔Q_{text}}+L_{Q↔Q_{text}} \end{array}$$

## 4. Experimental Results

### 4.1. Implementation Details

In our model, we employ the Swin-T [35] as the backbone. The backbone is pre-trained on the ImageNet-1k dataset with an image resolution of 224 × 224. For the ADE20K [10] and Cityscapes [11] datasets, the input images are cropped to sizes of 512 × 512 and 512 × 1024, respectively. Our implementation uses a batch size of 16 for the ADE20K dataset and 10 for the Cityscapes dataset. Our model is built with the PyTorch (1.10.1) [39] framework and the Detectron2 (v0.6) [40] library. We utilize the AdamW [41] optimization algorithm, setting the base learning rate to 0.0001 for the ADE20K dataset and to 0.00009 for the Cityscapes dataset.

### 4.2. Datasets

To evaluate the performance of our proposed model, we conduct experiments on two widely recognized datasets in computer vision: ADE20K [10] and Cityscapes [11]:

- ADE20K Dataset: This dataset is extensively used in research due to its rich annotations and diversity of scenes. It comprises over 20,000 images depicting a wide range of environments, each annotated at the pixel level for more than 150 object categories. Such detailed annotations make ADE20K particularly suitable for tasks like semantic and instance segmentation.
- Cityscapes Dataset: Designed specifically for urban street scene understanding, the Cityscapes dataset consists of 5000 images collected from 50 different cities. Each image is annotated with pixel-level labels for 19 semantic classes relevant to urban driving scenarios. The dataset is divided into a training set of 2975 images, a validation set of 500 images, and a test set of 1525 images.

For the evaluation metrics, we utilize three key measures standard in segmentation tasks: Mean Intersection over Union (mIoU) [42] for semantic segmentation is a metric that calculates the average overlap between the predicted segmentation and the ground truth across all classes. Panoptic Quality (PQ) [43] for panoptic segmentation provides a holistic evaluation by considering both the Segmentation Quality of “stuff” (background regions) and “things” (foreground objects). It is defined as PQ=SQ×RQ, where the Segmentation Quality (SQ) measures the average IoU of correctly matched segments, reflecting how precisely the model segments the objects. Recognition Quality (RQ) is the harmonic mean of precision and recall, indicating the model’s effectiveness in detecting and classifying objects correctly. Average Precision (AP) [44] for instance segmentation evaluates the model’s accuracy in detecting and delineating individual object instances, considering both localization and classification performance.

By employing these datasets and evaluation metrics, we provide a comprehensive assessment of our model’s performance across various segmentation tasks.

### 4.3. Experimental Results

#### 4.3.1. ADE20K

To evaluate the efficacy of our proposed model, we conduct experiments on the ADE20K dataset [10], with the results summarized in Table 1. This table compares our model against other leading universal segmentation models that have a similar number of parameters. All models, except kMaX-DeepLab [45], are trained on images with a resolution of 512×512. The input size of kMaX-DeepLab [45] is 1281×1281. An important aspect of our meta-architecture [5] is that components related to query–text contrastive learning are omitted during the inference phase. Consequently, when comparing the net parameter counts, we focus on the training phase—particularly between our model and EQO [5]. Additionally, the computational complexity, measured in GFLOPs (Giga Floating Point Operations), is calculated during the evaluation stage.

Our approach offers a significant reduction in parameter complexity, reducing the total number of parameters by 3.3 million compared to the baseline model [5]. In addition, our model achieves higher throughput during training, resulting in a faster training cycle. Especially in inference, we successfully reduce the computational costs by 11% in terms of GFLOPs, compared to EQO [5].

**Figure 4:**
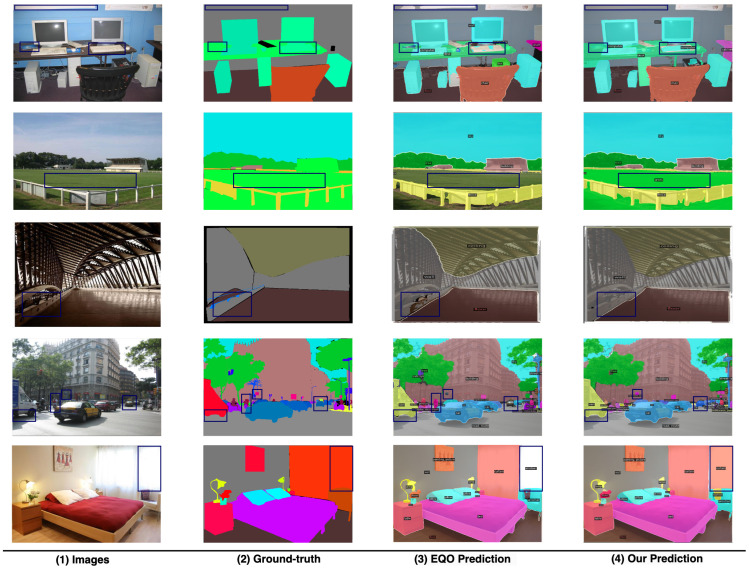
Panoptic Predictions Visualization on ADE20K val. Compared to our meta-architecture [5], our model achieves significant improvements in diminishing misclassification, capturing small objects, and outlining precise boundaries between different instances. The discrepancies in predictions are highlighted using blue rectangular boxes.

Notably, these gains in efficiency are accompanied by enhancements in performance. Our model demonstrates superior results in both semantic and panoptic segmentation tasks compared to the baseline [5]. Specifically, we observe a 0.9% increase in mean Intersection over Union (mIoU) and a 0.8% improvement in Panoptic Quality (PQ), while maintaining the same Average Precision (AP) score for instance segmentation. When compared to other universal segmentation models of a similar size [1,2,4], our model exhibits even greater performance advantages. For visual illustrations of our model’s predictions, please refer to Figure 4.

#### 4.3.2. Cityscapes

Table 2 presents a validation of our model’s performance across three tasks on the Cityscapes [11] dataset, comparing it with other competitive models in universal image segmentation. The training images for the models listed in Table 2 are cropped to a size of 512×1024, except for SeMask [49], which uses images of 768×768. Notably, our model achieves a reduction of 3.3 million parameters compared to its baseline [5] and outperforms it in instance segmentation by 0.5%. Additionally, it attains equal performance in semantic segmentation and comparable results in panoptic segmentation.

### 4.4. Ablation Study

The analysis of our model is performed using Swin-T backbone on the ADE20K [10] dataset.

#### 4.4.1. Ablation Studies on Each Presented Modules

To evaluate the contributions of each component in our model, we perform an ablation study by incrementally adding modules to our meta-architecture [5]. The results of this study are presented in Table 3.

The introduction of learnable task tokens leads to a significant increase in efficiency and an improvement of up to 1.2% in mean Intersection over Union (mIoU), while maintaining consistent Panoptic Quality (PQ) scores and comparable Average Precision (AP) scores. This indicates that learnable task tokens are more effective than predefined task tokens in capturing inter-task differences. During training, these tokens autonomously discern cross-task distinctions from image features and text queries, thereby enhancing segmentation performance.

Furthermore, the integration of our Adaptive Feature Alignment (AFA) module, designed to align text queries with image features, results in additional performance gains of 0.8% in panoptic segmentation and 0.2% in instance segmentation. These improvements support our assertion that AFA effectively bridges the cross-modality gap between the image and text branches. Moreover, the modality alignment achieved by AFA does not require large-scale encoders or extensive image–text paired datasets for training, underscoring the effectiveness of the class-specific means formulated by the AFA module.

#### 4.4.2. Ablation Studies on Learnable Task Tokens

In our approach, we utilize a learnable task token tailored for specific segmentation tasks to generate task-conditioned object queries. These object queries interact with image features within the transformer decoder module and are also linked to text queries through query–text contrastive learning. Through both interactions, the learnable task token captures inter-task distinctions from both image and text modalities. To further validate our design, we introduce two alternative variants of the original learnable task tokens. After selecting a learnable parameter $Q_{task}$ for a specific task, the first variant concatenates $Q_{task}$ with the supervisory text tokens used to generate text queries. This concatenated sequence is then input into the text encoder, allowing $Q_{task}$ to directly learn inter-task distinctions from the text queries via self-attention mechanisms. Subsequently, the updated $Q_{task}$ is separated from the text encoder’s output. We designate this output as learnable task token v0.1. However, as illustrated in Table 4, incorporating learnable task token v0.1 results in significant performance degradation across all three segmentation tasks. Specifically, the performance decline arises from integrating $Q_{task}$ into the text queries. Since text queries contain supervisory information derived from ground-truth labels, introducing a randomly initialized parameter $Q_{task}$ is equivalent to adding noise. Given that our text encoder is lightweight and lacks sufficient robustness, this disturbance diminishes the efficacy of text supervision in text queries, leading to the observed performance decline.

Next, we investigate a second alternative configuration, where a contrastive loss is computed between the original learnable task token $Q_{task}$ and the text queries. We refer to this setup as learnable task token v0.2, with results detailed in Table 4. Although v0.2 demonstrates improvements over v0.1, it still underperforms relative to our baseline model [5], particularly in panoptic segmentation. We attribute this shortfall to the absence of a large-scale text encoder. In this configuration, the text encoder must handle additional responsibilities: aggregating key semantic information and cross-task distinctions to guide the learnable task token via the contrastive loss, in addition to its primary role of generating text queries. This added complexity hampers the training process, resulting in degraded performance.

In contrast to the aforementioned configurations, our straightforward design of the learnable task token achieves substantial performance enhancements across various segmentation tasks without incurring additional computational costs. This highlights the efficacy of our approach in efficiently improving the model performance.

On the other hand, learnable task tokens capture intrinsic distinctions among segmentation tasks, making the model task-sensitive. As illustrated in Table 5, we train three task-specific learnable tokens $Q_{t1},Q_{t2}$, and $Q_{t3}$ for semantic, panoptic, and instance segmentation, respectively. We then examine how the model performs in inference when the chosen task token does not match the current task. When the task is panoptic segmentation, both $Q_{t1}$ and $Q_{t3}$ lead to considerable performance degradation in PQ, yet $Q_{t1}$ achieves a high $PQ^{St}$ score, reflecting its strong capability for recognizing amorphous “stuff” classes. In contrast, $Q_{t3}$ outperforms $Q_{t1}$ in terms of $PQ^{Th}$, demonstrating its effectiveness in modeling “thing” classes. Furthermore, because panoptic segmentation integrates the characteristics of semantic and instance segmentation, $Q_{t2}$ (trained for panoptic segmentation) performs well even when the current task is not panoptic. Specifically, when the task is instance segmentation, switching to $Q_{t1}$ causes an 8.1% drop in AP, whereas using $Q_{t2}$ only reduces AP by 0.2%. Similarly, in semantic segmentation, $Q_{t2}$ still delivers a competitive mIoU, whereas $Q_{t3}$ induces substantial performance loss. These findings validate the effectiveness of our learnable task tokens in capturing cross-task distinctions and highlight the task sensitivity of the resulting model.

#### 4.4.3. Ablation Studies on Cross-Modality Alignment

In our Adaptive Feature Alignment (AFA) module, we extract class-specific means from the largest image feature map generated by the pixel decoder. To investigate how the resolution of feature maps affects the AFA performance, we replace the largest feature map with the smallest one and conducted an experiment. This configuration is designated as AFA v0.1, and the corresponding results are presented in Table 6. The findings reveal that AFA v0.1 underperforms compared to our baseline model [5], highlighting the importance of generating class-specific means from a detailed, high-resolution image feature map for optimal effectiveness.

Furthermore, considering that object queries are linked to text queries through the query–text contrastive loss in our meta-architecture [5], we explore an alternative alignment strategy. This approach aims to bridge the image–text gap by computing the contrastive loss between object queries and class-specific means, referred to as AFA v0.2. The objective is to indirectly connect text queries with image features via object queries since both are involved in contrastive learning with object queries in this scenario. As demonstrated in Table 6, AFA v0.2 exhibits inferior performance compared to our model, particularly in semantic and panoptic segmentation tasks. These results suggest that direct alignment is more effective than indirect alignment, as the latter requires object queries to simultaneously align with both modalities. This dual alignment complicates the contrastive learning process and negatively impacts overall performance.

#### 4.4.4. Ablation Studies on Image–Text Contrastive Loss’s Weight

We analyze how varying the weight $\lambda_{m_{class}↔Q_{text}}$ of the image–text contrastive loss affects prediction accuracy. The experimental results are presented in Table 7, indicating that $\lambda_{m_{class}↔Q_{text}}=0.5$ yields the best overall performance.

## 5. Limitations

Our proposed Adaptive Feature Alignment (AFA) significantly enhances the model’s performance on the ADE20K [10] dataset. However, the improvements observed on the Cityscapes [11] dataset are comparatively smaller. This difference is attributed to the higher image resolution and the greater average number of instances in the Cityscapes dataset, indicating that our efficient Adaptive Feature Alignment experiences diminishing returns as the data complexity increases. Additionally, our universal image segmentation model currently requires separate training for each dataset, which limits its adaptability and flexibility in diverse applications. In future work, we aim to extend our model to support cross-dataset and cross-task image segmentation, thereby enhancing its versatility and broadening its applicability.

## 6. Conclusions

We presented Adaptive Feature Alignment (AFA) with a learnable task token to enhance universal image segmentation. By effectively capturing inter-task differences and efficiently aligning visual and textual modalities using class-specific means, our model improves performance while achieving complexity reductions. Experiments on ADE20K and Cityscapes demonstrate that AFA outperforms baselines in both efficiency and effectiveness, achieving state-of-the-art results among models with comparable parameters.

## Figures and Tables

**Table 1:** Image segmentation on ADE20K val with 150 categories. The single-scale mIoU is reported.

Method	Backbone	mIoU (s.s.)	PQ	AP	#Params	GFLOPs	Throughput
***Individual Training***							
Swin-UperNet [35,46]	Swin-T ^†^	46.1 *	-	-	60.0 M	236.0	-
Segmenter [47]	DeiT-B [48] ^†^	48.7	-	-	86.0 M	-	-
MaskFormer [1]	Swin-T ^†^	46.7	-	-	42.0 M	55.0	-
	R101	45.5	-	-	60.0 M	73.0	-
Mask2Former [2]	Swin-T ^†^	47.7	-	-	47.4 M	74.0	-
	R101	47.8	-	-	63.0 M	90.0	-
SeMask [49]	Swin-S ^‡^	45.9	-	-	56.0 M	63.0	-
kMaX-DeepLab [45]	R50	45.3	42.3	-	57.0 M	295.0	-
***Joint Training***							
OneFormer [4]	Swin-T ^†^	49.0	42.8	28.7	68.3 M	81.4	16.0 img/s
EQO [5]	Swin-T ^†^	49.2	43.6	29.3	63.4 M	81.4	16.5 img/s
Our Model	Swin-T ^†^	**50.1**	**44.4**	**29.3**	60.1 M	72.5	16.7 img/s

†: Backbone is pre-trained on ImageNet-1k; ‡: backbone is pre-trained on ImageNet-22k; and *: multi-scale mIoU. Numbers in bold represent the best performance in each metric.

**Table 2:** Image segmentation on Cityscapes val. The single-scale mIoU is reported.

Method	Backbone	mIoU (s.s.)	PQ	AP	Params	GFLOPs	Throughput
***Individual Training***							
Segmenter [47]	DeiT-B [48] ^†^	80.6	-	-	86.0 M	-	-
SETR-PUP [50]	ViT-L	79.3	-	-	318.3 M	-	-
Mask2Former [2]	Swin-T ^†^	**82.1**	63.9	39.7	47.4 M	-	-
	R101	80.1	62.4	38.5	63.0 M	-	-
SeMask [49]	Swin-S ^‡^	77.1	-	-	56.0 M	134.0	-
CMT-DeepLab-S [51]	Axial-R50 [52] ^‡^	81.4	64.6	-	95.0 M	396.0	-
***Joint Training***							
OneFormer [4]	Swin-T ^†^	80.7	64.9	41.9	68.3 M	168.2	6.6 img/s
EQO [5]	Swin-T ^†^	81.0	**65.6**	41.9	63.4 M	168.2	7.9 img/s
Our Model	Swin-T ^†^	81.0	65.3	**42.4**	60.1 M	148.5	8.1 img/s

†: Backbone is pre-trained on ImageNet-1k; ‡: backbone is pre-trained on ImageNet-22k. Numbers in bold represent the best performance in each metric.

**Table 3:** Ablation on each component.

	PQ	mIoU	AP
Baseline [5]	43.6	49.2	29.3
+Learnable Task Token	43.6	**50.4**	29.1
+AFA (Our model)	**44.4**	50.1	**29.3**

Numbers in bold represent the best performance in each metric.

**Table 4:** Ablation on Task Token Design.

	PQ	mIoU	AP
Baseline [5]	43.6	49.2	**29.3**
+Learnable Task Token v0.1	41.6	48.6	26.6
+Learnable Task Token v0.2	43.1	49.3	29.1
+Learnable Task Token	**43.6**	**50.4**	29.1

Numbers in bold represent the best performance in each metric.

**Table 5:** Ablation on varied task tokens.

Task Token Type	PQ	${\mathrm{PQ}}^{\mathrm{Th}}$	${\mathrm{PQ}}^{\mathrm{St}}$	mIoU	AP
Semantic ($Q_{t1}$)	36.8	31.8	**46.8**	50.1	21.2
Panoptic ($Q_{t2}$)	**44.4**	**43.5**	46.1	**50.3**	29.1
Instance ($Q_{t3}$)	29.0	42.3	2.4	25.1	**29.3**

Numbers in bold represent the best performance in each metric.

**Table 6:** Ablation on modality alignment.

	PQ	mIoU	AP
Baseline [5]	43.6	49.2	29.3
AFA v0.1	43.0	49.3	28.8
AFA v0.2	43.7	48.9	29.3
AFA (Our model)	**44.4**	**50.1**	**29.3**

Numbers in bold represent the best performance in each metric.

**Table 7:** Ablation on image–text contrastive loss’s weight.

	PQ	mIoU	AP
$\lambda_{m_{class}↔Q_{text}}=0.0$	43.6	**50.4**	29.1
$\lambda_{m_{class}↔Q_{text}}=0.2$	43.3	50.3	28.8
$\lambda_{m_{class}↔Q_{text}}=0.5$	**44.4**	50.1	**29.3**

Numbers in bold represent the best performance in each metric.

## Data Availability

The original data presented in the study are openly available in https://groups.csail.mit.edu/vision/datasets/ADE20K/index.html#Download and https://www.cityscapes-dataset.com.
